# E-cigarette use and smoking reduction or cessation in the 2010/2011 TUS-CPS longitudinal cohort

**DOI:** 10.1186/s12889-016-3770-x

**Published:** 2016-10-21

**Authors:** Yuyan Shi, John P. Pierce, Martha White, Maya Vijayaraghavan, Wilson Compton, Kevin Conway, Anne M. Hartman, Karen Messer

**Affiliations:** 1 Cancer Prevention and Control Program, Moores UC San Diego Cancer Center, 3855 Health Sciences Drive #0901, La Jolla, CA 92093-0901 USA; 2 Department of Family Medicine and Public Health, University of California, San Diego, 9500 Gilman Drive #0628, La Jolla, CA 92093-0628 USA; 3 Division of General Internal Medicine/San Francisco General Hospital, University of California, San Francisco, 1545 Divisadero St, San Francisco, CA 94115 USA; 4 National Institute on Drug Abuse, National Institutes of Health, 6001 Executive Boulevard, Bethesda, MD 20892-9589 USA; 5 Tobacco Control Research Branch, Behavioral Research Program, Division of Cancer Control and Population Sciences, National Cancer Institute, National Institutes of Health, 9609 Medical Center Drive, Bethesda, MD 20892-9761 USA

**Keywords:** Electronic cigarettes, Smoking cessation, Smoking reduction

## Abstract

**Background:**

Electronic cigarettes (e-cigarettes) are heavily marketed and widely perceived as helpful for quitting or reducing smoking intensity. We test whether ever-use of e-cigarettes among early adopters was associated with: 1) increased cigarette smoking cessation; and 2) reduced cigarette consumption.

**Methods:**

A representative cohort of U.S. smokers (*N* = 2454) from the 2010 Tobacco Use Supplement to the Current Population Survey (TUS-CPS) was re-interviewed 1 year later. Outcomes were smoking cessation for 30+ days and change in cigarette consumption at follow-up. E-cigarettes use was categorized as for cessation purposes or for another reason. Multivariate regression was used to adjust for demographics and baseline cigarette dependence level.

**Results:**

In 2011, an estimated 12 % of adult U.S. smokers had ever used e-cigarettes, and 41 % of these reported use to help quit smoking. Smokers who had used e-cigarettes for cessation were less likely to be quit for 30+ days at follow-up, compared to never-users who tried to quit (11.1 % vs 21.6 %; ORadj = 0.44, 95 % CI = 0.2–0.8). Among heavier smokers at baseline (15+ cigarettes per day (CPD)), ever-use of e-cigarettes was not associated with change in smoking consumption. Lighter smokers (<15 CPD) who had ever used e-cigarettes for quitting had stable consumption, while increased consumption was observed among all other lighter smokers, although this difference was not statistically significant.

**Conclusions:**

Among early adopters, ever-use of first generation e-cigarettes to aid quitting cigarette smoking was not associated with improved cessation or with reduced consumption, even among heavier smokers.

## Background

E-cigarettes have been intensively marketed since 2010 resulting in widespread awareness [1–5] and a fifteen-fold increase in sales between 2010 and 2014 [6, 7]. However, there is still little population-level evidence describing the association of cigarette smoking behaviors with e-cigarette use [8], and the role of e-cigarettes in tobacco control remains controversial [9, 10]. Given that few smokers quit successfully in any given year [11], it has been hypothesized that e-cigarette use may lead to reduced health risks, particularly for heavier smokers, by helping them to either quit cigarettes completely or to substantially reduce the number of cigarettes smoked each day [12]. This is counterbalanced with concern that widespread promotion of e-cigarettes may re-normalize smoking behavior and potentially increase tobacco use among youth [13–16]. The public health arguments against promoting e-cigarette use include that the health effects of long-term use are currently unknown [17], that e-cigarette aerosol contains similar numbers of fine particles, including nanoparticles, as cigarettes [13], and that aerosol may contain numerous toxicants including carcinogenic formaldehyde [13, 17, 18].

To date there is limited empirical evidence regarding the effect of e-cigarette use on cigarette smoking behavior, including smoking cessation [13]. A systematic review and meta-analysis by Kalkhoran and Glantz [8] summarized 20 studies and concluded that cigarette smokers who used e-cigarettes were 28 % less likely to quit smoking compared to those who did not use e-cigarettes. Among these 20 studies, 15 were cohort studies with follow-up. Only one published study used a national probability sample of the US: Grana et al. [19] reported that cigarette smokers in 2011 who used e-cigarettes were not more likely to quit or reduce cigarette consumption by the 1-year follow-up time-point.

In this paper we report findings from a longitudinal subsample of the large nationally representative 2010–2011 Tobacco Use Supplement to the Current Population Survey (TUS-CPS), fielded during a time of rapid uptake of e-cigarettes in the US population [20]. This survey assessed ever-use of e-cigarettes as well as reasons for use (for cessation, or for other purposes), and also use of pharmaceutical cessation aids. We compared the smoking characteristics between those who used e-cigarettes for quitting and those who used pharmaceutical aids for quitting. We used multivariate regression to examine the associations of ever use of e-cigarettes and use of pharmaceutical aids for smoking cessation and to reduce cigarette consumption, particularly among heavier smokers.

## Methods

### Data source

The core CPS is a labor force survey conducted monthly by the US Census Bureau from a nationally representative civilian, non-institutionalized adult sample, in which households are interviewed for 4 consecutive months, rested for 8 months, and then re-interviewed for another 4 months before exiting the sample. For the TUS portion of the survey, an interview is attempted with all eligible adults in the household. If unsuccessful in obtaining the self-interview, a proxy interview is sought. The TUS-CPS is funded by the U.S. National Cancer Institute and has been conducted since 1992 approximately every 3–4 years, to provide a nationally representative cohort of smokers and nonsmokers. The rotating panel design of the CPS provided an overlapped longitudinal sample of respondents to the May 2010 TUS survey (baseline) who were re-interviewed in May 2011 (follow-up; *n* = 28,153). This longitudinal subsample of the TUS-CPS uses self-report respondents only. Sample survey weights are provided to account for the complex sampling design, under coverage, and non-response in the overlap sample, allowing nationally representative estimates for the U.S. [21]. Data from the TUS-CPS contain no personal identifiers and are analyzed anonymously. The Institutional Review Board at the University of California, San Diego reviewed the study protocol and did not consider it to be human subjects research.

### Cigarette smoking measures

The TUS-CPS uses the standard national tobacco questions including “have you smoked at least 100 cigarettes in your entire life?” to identify ever smokers, and “do you now smoke cigarettes every day, some days, or not at all?” to identify current smokers. Average number of cigarettes smoked per day (CPD) was assessed. Change in consumption was computed as follow-up CPD minus baseline CPD, for continuing smokers. We classified those who smoked at least 15 CPD as heavier smokers, as in previous work [22].

### Cessation behaviors at follow-up

A quit attempt was identified with the question “during the past 12 months, have you stopped smoking for one day or longer because you were trying to quit smoking?” or “during the past 12 months, have you made a serious attempt to stop smoking because you were TRYING to quit – even if you stopped for less than a day?”. Those who smoked fewer than 12 days in the past 30 days were alternatively asked “during the past 12 months have you tried to quit smoking completely?” Respondents who reported smoking at baseline but said they now smoked not at all at follow-up were asked “about how long has it been since you completely quit smoking cigarettes”. We use 30+ days cessation at the follow-up survey as an early marker of successful cessation [23–25]. As a sensitivity analysis, we used 30+ days cessation on the longest quit attempt of the past 12 months [26, 27], which was assessed by “during the past 12 months, what is the longest length of time you stopped smoking because you were trying to quit”. These quit attempts lasting at least 30 days were analyzed in the sensitivity analysis.

### Ever use of e-cigarettes

At follow-up, ever-users of e-cigarettes were identified with the question “have you ever tried a product called an electronic or e-cigarette, such as ‘Smoking Everywhere’, ‘NJOY’, or other brands?” Use for quitting was assessed by the question “have you ever used e-cigarettes to help you quit smoking cigarettes or quit using other tobacco products?”

### Use of pharmaceutical assistance at most recent quit attempt

At follow-up, those who had made a quit attempt were asked about use of the following products on the most recent attempt: “a nicotine patch, a nicotine gum or nicotine lozenge, a nicotine nasal spray or nicotine inhaler?; a prescription pill, called Chantix or Varenicline?; a prescription pill, called Zyban, Bupropion, or Wellbutrin? another prescription pill?”.

### Other measures

We classified respondents into age groups (18–24 years, 25–34 years, 35–49 years, 50 years and older), as male or female, as non-Hispanic White or other race/ethnicity, and into four levels of educational attainment (less than high school, high school, some college, college and above). Using the question, “how soon after you wake up do you typically smoke your first cigarette of the day?” those who smoked within 30 min of waking were considered more dependent. Age of initiation <16 years classified a respondent as an early initiator, assessed with the question “how old were you when you first started smoking cigarettes fairly regularly”.

### Statistical methods

We described the demographic and smoking characteristics among those who made any quit attempts, ever used e-cigarettes, and ever used e-cigarettes for quitting. Chi-squared tests were conducted to compare differences by e-cigarette use status. We also compared smoking characteristics by e-cigarette and pharmaceutical aid use status. We used multivariate logistic regression to examine the relationship of 30+ day cessation at follow-up with ever use of e-cigarettes, and with use of pharmaceutical aids at last quit attempt, adjusted for baseline socio-demographic characteristics and nicotine dependence levels. E-cigarette use and use of pharmaceutical aids were coded as main effects in an additive model; these use categories are not mutually exclusive. Statistical significance was assessed at the two-sided 5 % level. All statistics were computed using TUS-CPS overlap sampling weights according to recommended procedures [21], using SAS 9.3 (SAS Institute) survey procedures.

## Results

In the longitudinal 2010–2011 TUS-CPS, there were 5255 baseline smokers who completed a self-reported interview. Follow- up information was available one year later on 3305 (62.9 %), however, only 2454 (46.7 %) completed the follow-up interview themselves (rather than by proxy) and thus provided a detailed quitting history. These 2454 baseline current smokers comprised the current study sample.

### Characteristics of the baseline sample

Table 1 summarizes the demographic and smoking characteristics of the cohort as well as quit attempts and e-cigarette use status. Of the baseline cigarette smokers (*N* = 2454), 43.6 % made a quit attempt during the one-year follow-up period (Table 1) and this varied inversely with age (52.1 % to 40.0 %, youngest to oldest). Lighter smokers were more likely to have made a quit attempt than heavier smokers (48.7 % vs 37.3 %); those who smoked within 30 min of waking were less likely to make a quit attempt than those who did not (40.6 % vs 45.3 %).

**Table 1:** Past year quit attempts and ever use of e-cigarettes, by demographic characteristics, in a nationally representative cohort of U.S. smokers; TUS-CPS 2010–11 longitudinal sample

	*N*	(%)	Past- year quit attempt (%)	Ever use of e-cigarettes for any reason (%)	Ever use for quitting (%)
Overall	2454	(100 %)	43.6	12.2	5.0
Age***					
18–24	117	(13.3 %)	52.1	21.7	6.7
25–34	400	(18.0 %)	45.5	14.1	4.8
35–49	818	(30.3 %)	43.4	10.4	5.0
50 and above	1119	(38.3 %)	40.0	9.4	4.6
Gender					
Female	1306	(46.6 %)	47.6	12.0	5.6
Male	1148	(53.4 %)	40.2	12.4	4.6
Race/Ethnicity***					
Non-Hispanic White	2001	(76.2 %)	43.4	13.8	5.9
Other	453	(23.8 %)	44.3	7.1	2.4
Education**					
Less than high school	383	(17.6 %)	42.6	7.4	2.6
High school	1005	(40.1 %)	42.5	12.0	4.7
Some college	740	(30.4 %)	47.0	13.9	6.4
College graduate	326	(11.9 %)	40.5	15.6	6.4
Cigarettes/day (cpd)***					
< 15	1244	(53.5 %)	48.7	9.9	3.8
15+	1175	(46.5 %)	37.3	15.1	6.6
Smoking < 30 min of waking					
No	1514	(64.4 %)	45.3	11.5	4.6
Yes	890	(35.6 %)	40.6	13.6	6.1
Smoking before age 16***					
No	2028	(83.3 %)	43.5	11.5	4.2
Yes	426	(16.7 %)	44.2	16.0	9.4

Note: *P*-values are from a chi-squared test across categories for differences in ever-use of e-cigarettes. Percentages are weighted to be representative of the U.S. population

**p < 0.01, ***p < 0.001

### Ever use of e-cigarettes

Of US smokers, an estimated 12.2 % reported ever having used e-cigarettes in 2011 (Table 1). E-cigarette use was higher among younger age groups (21.7 % for those aged 18–24 years vs 14.1 % for ages 25–34 years, and lower percentages in older age groups), and there was no gender difference. Ever-users were more likely to be non-Hispanic White than from other race-ethnic groups (13.8 % vs 7.1 %). Ever use increased with attained educational level, from 7.4 % to 15.6 %. Heavier smokers were more likely to have used than lighter smokers (15.1 % vs 9.9 %) and ever-users were more likely to have initiated before age 16 years than never-users (16.0 % vs 11.5 %).

Overall, 41.3 % of those who had ever used e-cigarettes said the reason for use was to help quit smoking. This percentage varied by age from a low of 31.0 % for 18–24 years old users to almost half of ever-users who were over age 35 years. Non-Hispanic whites were more likely to have used e-cigarettes for quitting than were other race-ethnic groups (42.5 % vs 33.6 %), as were those with at least some college compared to those with lesser education. Lighter smokers were less likely than heavier smokers to have used for quitting. Those who initiated smoking under age 16 years were more likely to have used e-cigarettes for quitting than those who initiated later (58.9 % vs 36.4 %). (Table 1).

### Pharmaceutical aid use on most recent quit attempt

Of those with a quit attempt during the year, 20.1 % reported using NRT and an additional 10.4 % reported using Chantix. Only 3.1 % reported using Zyban and 0.5 % reported using any other pharmaceutical aid. Among smokers who used any pharmaceutical aid, 16.0 % had quit for 30+ days at follow-up, with little difference across type of pharmaceutical aid used. Accordingly, we used the combined category “any pharmaceutical use” on the most recent quit attempt for the remainder of this paper.

### Baseline smoking characteristics by product use

Table 2 presents nicotine dependence characteristics by e-cigarette and pharmaceutical aid use status, among smokers with a quit attempt. As many smokers used multiple cessation aids, the e-cigarette and pharmaceutical aid use categories are not mutually exclusive. Smokers who either used a pharmaceutical aid on their last quit attempt or had ever used an e-cigarette to quit were more likely to be higher intensity smokers than those who did not (14.1 CPD, pharmaceutical aid users; 14.9 CPD, e-cigarette ever-users who used for quitting; <12 CPD, other categories). They were more likely to smoke their first cigarette within the first 30 min after waking and more likely to have initiated smoking by age 15 years than those who did not use either e-cigarettes or pharmaceutical aids to quit.

**Table 2:** Baseline smoking characteristics by product use, among those who made a quit attempt; TUS-CPS 2010–11 longitudinal sample

	E-cigarette use status			Pharmaceutical aid use status	
	Ever used e-cigarettes for quitting	Ever used e-cigarettes for other purposes	Never used e-cigarettes	Used pharma aids at last quit attempt	No use of pharma aids at last quit attempt
Sample size	82	58	936	356	720
Cigarettes smoked/day (CPD)	14.9	11.5	11.8	14.1	11.1
Time to 1^st^ cigarette < 30 min from waking (%)	44.8	38.7	31.6	41.7	29.5
Duration of Cigarette Use (years)	23.5	20.2	24.6	26.7	23.2
Smoking Before Age 16 (%)	29.3	20.9	15.4	24.5	13.7

Note: E-cigarette use status and pharmaceutical aid use status are not mutually exclusive. All data weighted to be representative of the U.S. population

### Quit for 30+ days at follow-up, among those who tried to quit

**Fig. 1:**
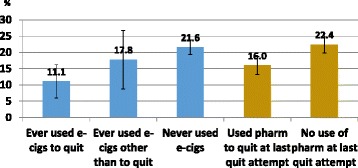
Percentage of smokers with 30+ day cessation at follow-up, among those who made a quit attempt

Among smokers who tried to quit, 21.6 % of those who had never used e-cigarettes were quit for 30+ days at follow-up, similar to the 22.4 % quit rate among those who did not use a pharmaceutical aid in their last quit attempt (Fig. 1). Ever use of an e-cigarette to quit was associated with a lower success rate (11.1 %, Fig. 1) than those who had not used an e-cigarette, and this difference was significant in an adjusted logistic regression model (Table 3, OR_adj_ = 0.4, 95 % C.I.: 0.2–0.8). Similarly, smokers who used a pharmaceutical aid at last quit attempt had a lower success rate (16.0 %, Fig. 1) than those who did not (Table 3, OR_adj_ = 0.7, 95 % C.I.: 0.5–0.9). The success rate among those who ever used e-cigarettes for cessation was observed to be substantially lower than among those who used pharmaceutical aids at the last quit attempt (Fig. 1). In a sensitivity analysis, the same model using as outcome any cessation of 30+ days during the past year had consistent results (Table 4) and the cessation effect associated with use of e-cigarettes was significantly less than for use of pharmaceutical cessation aids.

**Table 3:** Logistic regression for 30+ day cessation at follow-up, among those who made a quit attempt; TUS-CPS 2010–11

Variable	Percent attaining outcome	Adjusted odds ratio	95 % CI
E-cigarette ever use status			
Never used	34.9	Reference	
Ever used to quit	17.3	0.4	0.2–0.8**
Ever used other than to quit	30.4	0.7	0.4–1.5
Pharmaceutical aid used for last quit attempt			
No	34.7	Reference	
Yes	29.5	0.7	0.5–0.9**
Age			
18–24	40.6	Reference	
25–34	38.8	1.5	0.9–2.6
35–49	30.2	0.7	0.4–1.2
50+	29.3	0.8	0.5–1.4
Sex			
Female	33.9	Reference	
Male	32.4	1.1	0.8–1.4
Race/Ethnicity			
Non-Hispanic White	32.6	Reference	
Other	35.0	1.0	0.7–1.3
Education			
Less than high school	20.9	Reference	
High school	32.1	2.2	1.4–3.4***
Some college	40.5	4.0	2.6–6.1***
College gradate	34.4	3.2	1.9–5.5***
Cigarettes smoked per day			
< 15	36.2	Reference	
15+	28.9	1.0	0.7–1.2
Time to 1st cigarette < 30 min from waking			
No	34.2	Reference	
Yes	31.5	0.9	0.7–1.2
Smoking initiated before age 16 years			
No	32.3	Reference	
Yes	37.4	1.2	0.8–1.7

**p < 0.01, ***p < 0.001 All data is weighted to be representative of the U.S. population

**Table 4:** Sensitivity analysis: logistic regression for any 30+ day cessation during the past year, reported at follow-up, among those who made a quit attempt; TUS-CPS 2010–11

Variable	Percent attaining outcome	Adjusted odds ratio	95 % CI
E-cigarette ever use status			
Never used	21.6	Reference	
Used to quit	11.1	0.3	0.2–0.5***
Used other than to quit	17.8	0.7	0.4–1.3
Pharmaceutical aid used for last quit attempt			
No	22.4	Reference	
Yes	16.0	0.8	0.6–1.1
Age			
18–24	22.6	Reference	
25–34	31.3	0.9	0.5–1.4
35–49	16.1	0.6	0.4–1.1
50+	17.5	0.6	0.4–1.0
Sex			
Female	20.0	Reference	
Male	21.1	1.0	0.8–1.2
Race/Ethnicity			
Non-Hispanic White	20.9	Reference	
Other	19.2	1.2	0.9–1.6
Education			
Less than high school	9.1	Reference	
High school	17.6	2.0	1.4–2.7***
Some college	28.6	2.7	1.9–3.8***
College gradate	24.5	2.2	1.4–3.5***
Cigarettes smoked per day			
< 15	22.5	Reference	
15+	17.8	0.8	0.6–1.0*
Time to 1st cigarette < 30 min from waking			
No	22.2	Reference	
Yes	17.4	1.1	0.8–1.4
Smoking initiated before age 16 years			
No	20.4	Reference	
Yes	21.3	1.4	1.1–1.8*

*p < 0.05, ***p < 0.001 All data is weighted to be representative of the U.S. population

### Change in cigarette smoking intensity over 12 months

**Fig. 2:**
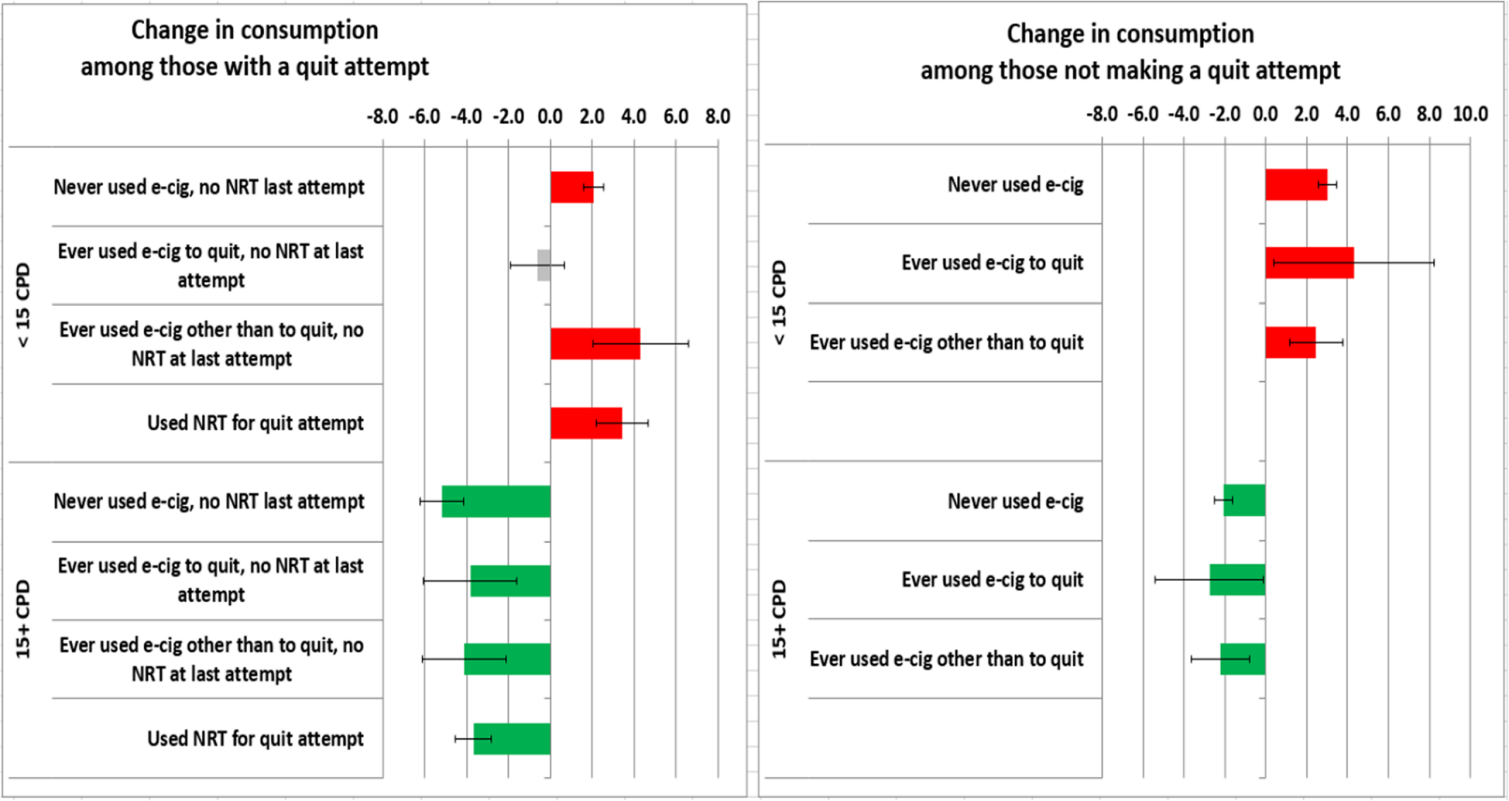
Changes in smoking intensity among smokers by product, baseline consumption level and reported cessation attempts

Figure 2 presents change in cigarette consumption for lighter and heavier smokers by categories of use of e-cigarettes and pharmaceutical aids, separately for those who made a quit attempt and who did not. Among heavier smokers (15+ CPD) who did not make a quit attempt, all decreased their consumption over the year by 2.1 to 2.8 CPD, a regression-to-the-mean phenomenon. Heavier smokers who reported a quit attempt during the year decreased their consumption by 3.7–5.2 CPD and the largest decline was observed among those who reported no use of either e-cigarettes or pharmaceutical aids, although the difference between these groups was not significant. Among lighter smokers (<15 CPD) who did not make a quit attempt, cigarette smoking consumption increased over the year (+2.5–4.3 CPD), again as expected from regression-to-the-mean. A similar pattern was seen among light smokers who made a quit attempt (+2.0–4.3 CPD), with the exception of those who ever-used e-cigarettes to quit. This group did not increase cigarette smoking consumption over the one-year follow-up period (change = −0.6 CPD, 95 % CI = −1.9–0.7), although again the difference between groups was not statistically significant. Multivariate linear regression adjusted for baseline characteristics provides consistent results (available upon request).

## Discussion

This is the first longitudinal study to use a nationally representative sample of U.S. smokers to provide evidence regarding the impact of e-cigarettes on smoking behavior at the U.S. population level. This study, conducted before e-cigarettes were widely used, did not find evidence that the ever-use of e-cigarettes as a cessation aid among these early adopters was associated with increased 30+ day smoking cessation at one-year follow-up. Further, reported ever use of e-cigarettes by heavier smokers was not associated with reduced cigarette consumption at follow-up. However, among lighter smokers, those who had ever used e-cigarettes for cessation had stable consumption levels, whereas all other light smokers showed increased consumption at follow-up, although these differences were not statistically significant.

In this study, those who had ever used e-cigarettes to quit or had used pharmaceutical aids on their most recent quit attempt equally appeared to be heavier, more dependent smokers. Such a finding is well known for pharmaceutical aids [28]. Thus, it was expected that success rates for e-cigarette users and for pharmaceutical aid users might be less than for non-users in this population study, as we found to be the case in both adjusted and unadjusted analysis. However, the data also suggest that cessation rates among those who had used e-cigarettes for quitting may be lower than cessation rates seen with use of a pharmaceutical aid.

While our study is the first which can provide nationally representative estimates for the U.S and focuses on early adopters who used first generation e-cigarettes, it supports results from the other published U.S. population-based longitudinal cohort study [19], as well as the summary results in meta-analysis across all 20 studies published as of this writing [8]. Most recently, a Canadian study of smokers enrolled in cessation assistance in the primary care setting showed association of e-cigarette use with poor cessation outcomes [29], consistent with our results. In contrast, relying on samples in two U.S. metropolitan areas Biener et al. [23] reported a strong association of smoking cessation with daily use of e-cigarettes for 30+ days, as compared to non-users. However, most (66 %) of those reporting daily e-cigarette use had adopted e-cigarettes because they wanted to quit smoking, whereas non-users did not report the same motivation, and the resulting confounding by reverse causation can account for the reported association. Indeed, to control for such confounding in our study, we compared use and non-use of cessation aids only among those who had tried to quit, as in prior work [26, 27, 30, 31]; without this careful choice of comparison group, we are able to replicate a spurious association which is similar in magnitude to that reported by Biener et al. [23] (data available upon request). However, our study is unable to address whether extensive and heavy use of e-cigarettes might facilitate cessation, as we were unable to find such users in the national population. As e-cigarette use moves beyond early adopters into the population and product innovation continues, such heavy use may become more common allowing the association with cessation to be further tested [6, 32]. Our observation that use of e-cigarettes by light smokers appeared to prevent increased consumption by follow-up was not hypothesized prior to this study. Thus, it is a hypothesis-generating observation in need of replication.

Our study has a number of limitations. The survey was conducted when e-cigarette use was uncommon but rapidly increasing in the US. Accordingly, the e-cigarette question at follow-up only sought information on ever-use, as well as whether the use was for cessation. Ideally, the usage report would have been linked directly to the most recent quit attempt. However, in 2009 ever use of e-cigarettes among smokers was less than 2 %, and 40 % of ever-users also reported past 30-day use [4, 33]. Thus we expect that most reported use fell within the past year, as did the quit attempt. However, these findings need to be replicated in other nationally representative longitudinal studies. Although the diversity of e-cigarette products was much smaller in 2010–2011 than at present [32], the TUS-CPS treated e-cigarettes as a single homogeneous product and there was no information on intensity of use, whereas others have noted substantial variability in nicotine levels, patterns of use and customer experiences [34–36]. In the future it will be important to investigate patterns of cigarette use among sub-populations of e-cigarette users.

## Conclusions

Among early adopters of first generation products, ever-use of e-cigarettes to aid cigarette smoking cessation was not associated with either improved cessation outcomes or with reduced cigarette consumption, in the U.S. population. The data also suggested that cessation rates among those who had used e-cigarettes for quitting may be lower than cessation rates seen with use of a pharmaceutical aid in the U.S. Thus, this study suggests that it is premature to conclude that e-cigarettes will be helpful to smokers who make a quit attempt. It also seems premature to conclude that e-cigarettes will be an effective way for smokers, especially heavy smokers, to reduce the number of cigarettes that they smoke each day. Given the intensive marketing and rapid increase in use of e-cigarettes, it will be important to study advertising content as well as smokers’ attitudes and beliefs regarding these products, in order to assess the role of e-cigarettes in changing smoking behavior.
